# The Tubular Penetration Depth and Adaption of Four Sealers: A Scanning Electron Microscopic Study

**DOI:** 10.1155/2017/2946524

**Published:** 2017-12-31

**Authors:** Huan Chen, Xinyuan Zhao, Yu Qiu, Dengyou Xu, Li Cui, Buling Wu

**Affiliations:** ^1^Shenzhen Stomatological Hospital, Southern Medical University, Shenzhen 518000, China; ^2^Department of Stomatology, Nanfang Hospital, Southern Medical University, Guangzhou 510000, China; ^3^Stomatological Hospital, Southern Medical University, Guangzhou 510280, China; ^4^Department of Oral and Maxillofacial Surgery, The First Affiliated Hospital of Fujian Medical University, Laboratory of Facial Plastic and Reconstruction of Fujian Medical University, Fuzhou 350000, China; ^5^UCLA School of Dentistry, Los Angeles, CA 90095, USA

## Abstract

*Background. *The tubular penetration and adaptation of the sealer are important factors for successful root canal filling. The aim of this study was to evaluate the tubular penetration depth of four different sealers in the coronal, middle, and apical third of root canals as well as the adaptation of these sealers to root canal walls.* Materials and Methods. *50 single-rooted teeth were prepared in this study. Forty-eight of them were filled with different sealers (Cortisomol, iRoot SP, AH-Plus, and RealSeal SE) and respective core filling materials. Then the specimens were sectioned and scanning electron microscopy was employed to assess the tubular penetration and adaptation of the sealers.* Results*. Our results demonstrated that the maximum penetration was exhibited by RealSeal SE, followed by AH-Plus, iRoot SP, and Cortisomol. As regards the adaptation property to root canal walls, AH-Plus has best adaptation capacity followed by iRoot SP, RealSeal SE, and Cortisomol.* Conclusion*. The tubular penetration and adaptation vary with the different sealers investigated. RealSeal SE showed the most optimal tubular penetration, whereas AH-Plus presented the best adaptation to the root canal walls.

## 1. Introduction

Removing the infectious materials in the root canal completely and leak-proof root filling play crucial roles in preventing reinfection after root canal treatment (RCT) [1]. However, tiny amount of infectious substance might still remain in the root canal system even using the best root canal disinfection regiment. The remaining bacterial and their products as well as dentin chip have the tendency to enter the dentinal tubule due to its permeability and the siphon effect. In addition, the adhesive capacity of bacterial is beneficial to its adhesion with the surface of peritubular dentin and intertubular dentin [2]. These conditions provide a good environment for bacterial reproduction and proliferation, resulting in the reinfection of root canal system. Therefore, obturation of the complex root canal system using filling materials with superior sealing capacity, which can embed the remaining bacterial and block the transportation between root canal and apical tissues, is an effective strategy to solve this problem.

Previously too much unnecessary emphasis has been placed on whether the filling has reached the radiographic apex or not [3]. Three-dimensional obturation of root canal is important for ensuring the long term success of RCT [4]. Except for coronal and apical microleakage, the microgap between sealer and the root canal wall as well as its tubule penetration depth is also a key factor associated with the clinical outcome of RCT. Good adaptations between sealer and root canal wall can not only reduce the chance of microleakage, but also increase the breaking strength of root canal significantly [5–7]. The advantages of deep tubule penetration depth are obvious. Firstly, it enhances the contact area between root fillings and dentin, which can increase the sealing capacity of the whole root canal system [8]. In addition, it might prevent the bacteria from entering the dentin tubule and its antimicrobial effect will increase when in closer contact with the microbes [9, 10]. Moreover, deep sealer penetration also raises the fracture resistance of root canal. Therefore, tubular penetration depth and adaptation are two crucial properties for an ideal sealer.

To the best of our knowledge, few studies have comprehensively evaluated the penetration depth and adaptation of current available sealers. Therefore, this study was designed to compare the penetration depth and adaptation of four general types of sealers including Cortisomol (zinc oxide-based sealer), iRoot SP (calcium silicate-based sealer), AH-Plus (epoxy resin-based sealer), and RealSeal SE (self-adhesive methacrylate resin-based sealers).

## 2. Materials and Methods

### 2.1. Sample Collection

This study was approved by Institutional Review Board of Nanfang Hospital, Southern Medical University. Fifty caries free and single-rooted permanent mandibular second premolars which were extracted for orthodontic reasons were included. Informed consent for extraction of teeth to be used for this study was obtained from each individual. All the teeth had intact roots and apical foramen. In addition, the roots were similar in both size and length. Teeth with root canal fillings, fracture, and resorption were rejected. The teeth were stored in distilled water after removing extraneous tissues and calculus.

### 2.2. Specimen Preparation

The coronal portions of teeth were sectioned to standardize the size of the root canal length at 14 mm. The working length (WL) was determined by introducing a size 10 K-file (Dentsply Maillefer, Ballaigues, Switzerland) into the canal until the file was just visible at the apical foramen under magnification and then subtracting 1 mm. The specimens were prepared using the crown-down technique with ProTaper rotary nickel-titanium instruments (Dentsply Maillefer). The apical preparation was done up to size F3. After the use of each instrument, canals were irrigated with 4 mL of 2.5% sodium hypochlorite (NaOCl, Sigma-Aldrich, St. Louis, MO, USA), followed by 3 mL of 17% ethylenediaminetetraacetic acid (EDTA, Sigma-Aldrich) and finally washed with 5 mL of distilled water. The root canals were dried using sterile absorbent paper points (Dentsply Maillefer).

Two specimens were randomly selected and served as the blank control group to verify the absence of smear layer on the dentinal walls. The remaining 48 samples were randomly divided into 4 groups (*n* = 12): group A: gutta-percha (Dentsply Maillefer)/Cortisomol (Pierre Roland, Merignac Cedex, France); group B: gutta-percha (Dentsply Maillefer)/iRoot SP (Innovative BioCeramix, Vancouver, BC, Canada); group C: gutta-percha (Dentsply Maillefer)/AH-Plus (Dentsply Maillefer); group D: Resilon (Dentsply Maillefer)/RealSeal SE (SybronEndo Corporation, Orange, CA, USA). All the four sealers were mixed according to the manufacturers' recommendations and placed into the root canals using a Lentulo spiral #25 (Dentsply Maillefer) in low-speed handpiece (Sirona Dental Systems INC, Long Island City, NY, USA). Lateral condensation filling technique was employed for root canal fillings and then the crown sections of all specimens were sealed off using resin composite (SDS Kerr, Orange, CA, USA). The samples were stored in 100% humidity at 37°C for 10 days to allow the sealers to set.

The filled roots were sectioned at 2, 5, and 8 mm from the apex by using bone chisel (DT Surgical Instruments, Murrieta, CA, USA), which represented the apical third, middle third, and coronal third, respectively. The specimens were washed by 17% EDTA for 2 min, followed by 5.25% NaOCl for 3 min and finally distilled water for 5 min.

### 2.3. Scanning Electron Microscopy Analysis

The samples were dehydrated, mounted on an aluminum stub, sputter coated with gold, and observed under scanning electron microscopy (SEM, S-3000 N, Hitachi, Japan). SEM was initially performed at 20 kV accelerating voltage with 500x magnification. Then tubule penetration depth of the sealers as well as the width between sealer and root canal walls was assessed directly by quantitative measures with 2000x magnification. The most representative of root-sealer interface for each section (apical third, middle third, and coronal third for each specimen) was selected; then the minimum and maximum depth of sealer penetration in the tubules was measured. The quality of sealer adaptation to the intracanal dentine was examined and calculated at four directions (buccal, lingual, mesial, and distal) for each section. To avoid examiner bias, the data were recorded by two independent, double-blind researchers.

### 2.4. Statistical Analysis

The data were expressed as the mean ± standard deviation and analyzed by the One-Way ANOVA and Student-Newman-Keuls test using SPSS v21.0 software (SPSS Inc., Chicago, IL, USA). *p* values < 0.05 were regarded to be statistically significant.

## 3. Results

### 3.1. The Dentinal Tubules Were Open following Root Canal Preparation

**Figure 1:**
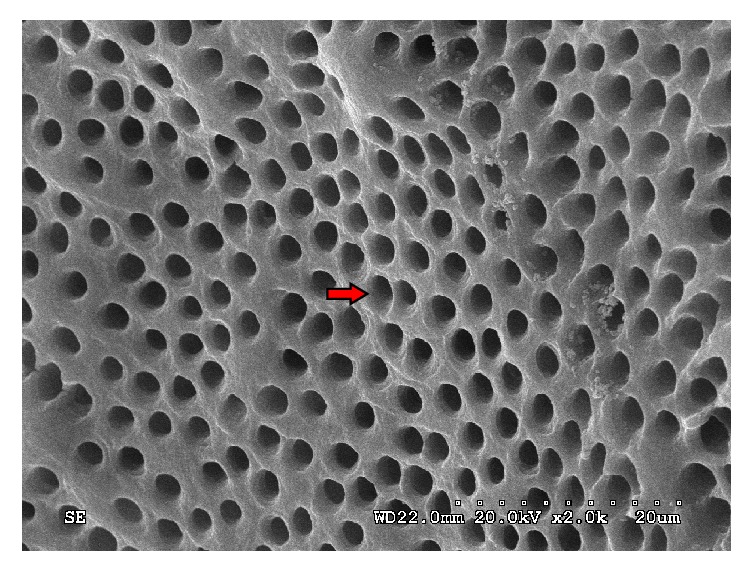
Root canal dentine surface was smooth and most smear layer was removed. In addition, the dentinal tubules were open. The red arrow indicates the openings of the dentinal tubules.

The SEM results of control group showed that the dentin surface of root canal was smooth and most smear layer was removed. In addition, the dentinal tubules were open. Although some debris was observed around the tubules, it did not obstruct the tubules (Figure 1).

### 3.2. Comparison of the Penetration Depth Capacity of Four Sealers

**Figure 2:**
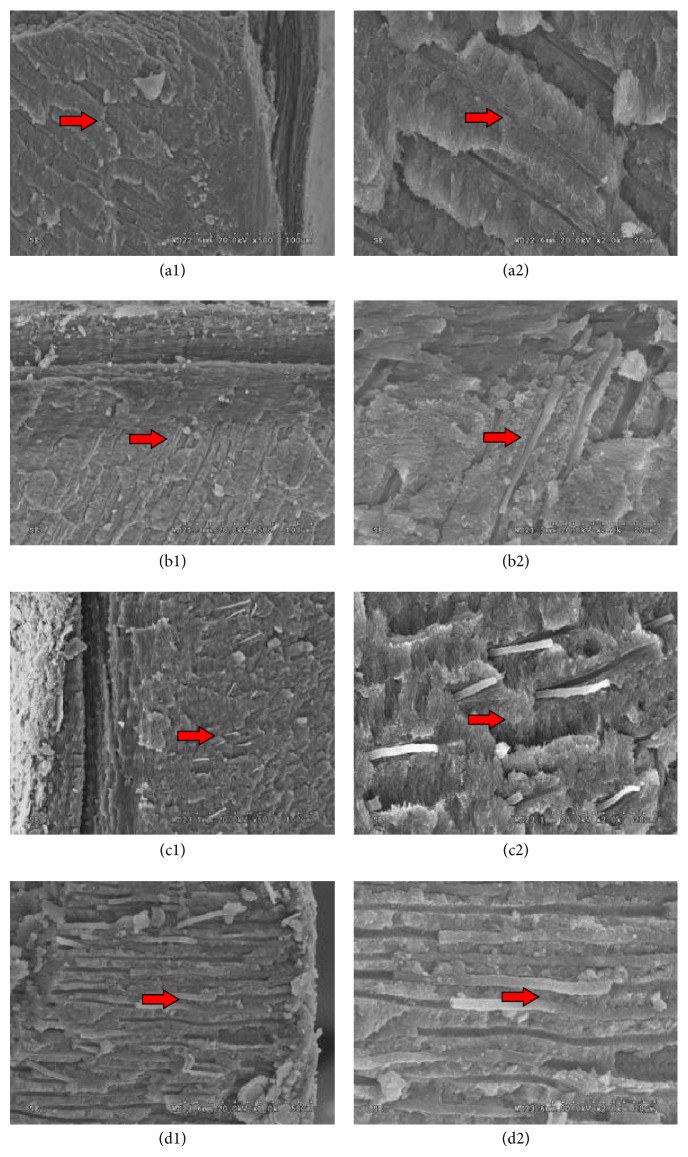
The tubular penetration depth of sealers. Many gaps could be found between the Cortisomol and the canna walls (Figures 2(a1) and 2(a2)). iRoot SP was cylinder-shaped and partially homogenous. There were some gaps between iRoot SP and dentinal tubules walls (Figures 2(b1) and 2(b2)). AH-Plus was spherical and homogenous. There was a good connection between the fillings and dentine tubules and little gaps were in existence (Figures 2(c1) and 2(c2)). RealSeal SE was cylinder-shaped and most homogeneous. It had a tight junction with tubules walls and no obvious gaps were observed (Figures 2(d1) and 2(d2)). The red arrows indicate the area that was zoomed in.

The amount of Cortisomol sealer that infiltrated in the dentine tubules was relatively scarce. Also, the sealer had a granular-like appearance and a loose connection with dentinal tubules. Many gaps could be found between the sealer and the walls dentinal tubules (Figures 2(a1) and 2(a2)). As regards the amount of sealer entering into the dentine tubules, iRoot SP performed better than Cortisomol. The fillings were cylinder-shaped and partially homogenous. The filling thickness was various and the filling near the tubule openings was thicker. There were some gaps between iRoot SP and dentinal tubules walls (Figures 2(b1) and 2(b2)). AH-Plus had better performance than Cortisomol and iRoot SP. The sealer was spherical and homogenous. There was a good connection between the fillings and dentine tubules and little gaps were in existence (Figures 2(c1) and 2(c2)). RealSeal SE performed best among the four sealers investigated. The fillings were cylinder-shaped and most homogeneous. RealSeal SE had a tight junction with tubules walls and no obvious gaps were observed (Figures 2(d1) and 2(d2)).

**Figure 3:**
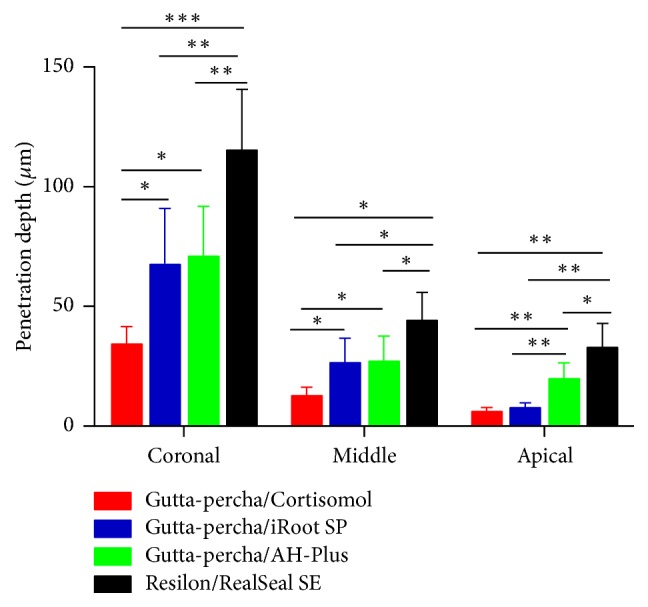
Comparison of the penetration depth of four sealers (^*∗*^*p* < 0.05, ^*∗∗*^*p* < 0.01, and ^*∗∗∗*^*p* < 0.001).

The penetration depth of four sealers in the dentinal tubules in the coronal, middle, and apical third of the root canal walls has been summarized in Table 1. The penetration depth of RealSeal SE was significantly deeper than the other three sealers in the coronal, middle, and apical third of the root canal (^*∗*^*p* < 0.05, ^*∗∗*^*p* < 0.01, and ^*∗∗∗*^*p* < 0.001). AH-Plus performed better than iRoot SP at the apical third of the root canals (^*∗∗*^*p* < 0.01), whereas no significant difference was found between these two sealers regarding their tubule penetration depth in the coronal and middle third of the root canals. The tubule penetration depth of AH-Plus was deeper than Cortisomol in all three parts of the root canals especially in the apical third (^*∗*^*p* < 0.05, ^*∗∗*^*p* < 0.01). iRoot SP performed better than Cortisomol in the coronal and middle third (^*∗*^*p* < 0.05) but not in the apical third (Figure 3).

### 3.3. Comparison of the Adaptation Capacity of Four Sealers

**Figure 4:**
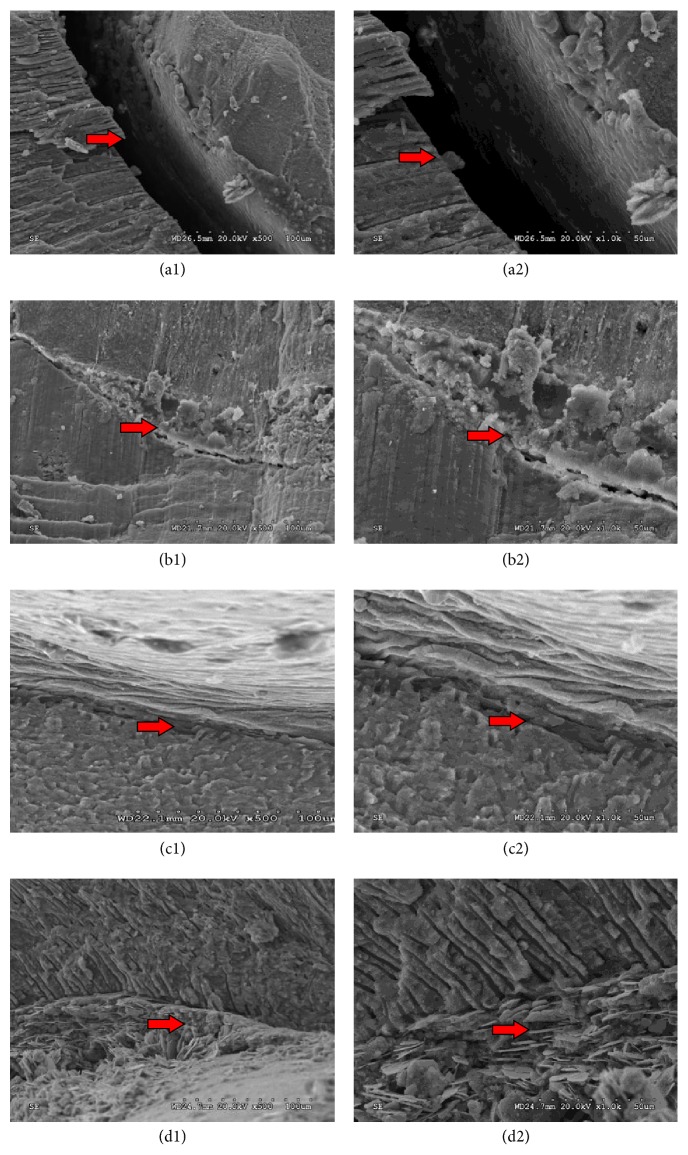
The gap width between sealer and root canal walls. There were large gaps between Cortisomol and root canal dentin (Figures 4(a1) and 4(a2)). iRoot SP had connections between both core fillings and root canal walls; however, gaps were still in existence (Figures 4(b1) and 4(b2)). Close junctions were found between AH-Plus and root canal dentin. In addition, the interface was streamline (Figures 4(c1) and 4(c2)). Similarly, RealSeal SE was connected with core fillings and root canal walls, but gaps were partly observed (Figures 4(d1) and 4(d2)). The red arrows indicate the area that was zoomed in.

There were large gaps between Cortisomol and root canal dentin. The sealer was attached to the core fillings, which looked like aggregation of many fusion spheres (Figures 4(a1) and 4(a2)). iRoot SP had connections between both core fillings and root canal walls. However, gaps were still in existence (Figures 4(b1) and 4(b2)). Close junctions were found between AH-Plus and root canal dentin. In addition, the interface was streamline (Figures 4(c1) and 4(c2)). Similarly, RealSeal SE was connected with core fillings and root canal walls, but gaps were partly observed (Figures 4(d1) and 4(d2)).

**Figure 5:**
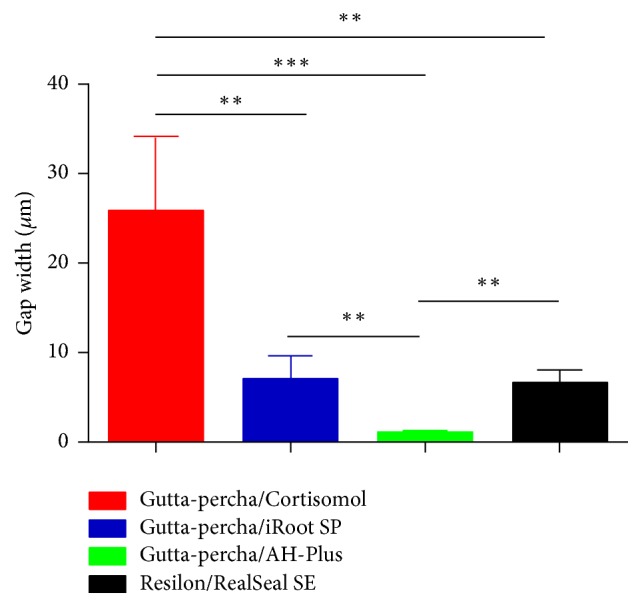
Comparison of the adaptation capacity of four sealers (^*∗∗*^*p* < 0.01, ^*∗∗∗*^*p* < 0.001).

The width of gaps between the sealers and apical third of the root canal walls were summarized in Table 2. The gap between Cortisomol and root canal wall in the apical third was significantly larger than the other three sealers (^*∗∗*^*p* < 0.01, ^*∗∗∗*^*p* < 0.001). AH-Plus had the smallest gaps and performed best among four materials (^*∗∗*^*p* < 0.01, ^*∗∗∗*^*p* < 0.001). Statistical significance was not detected between RealSeal SE and iRoot SP about the gaps between the sealers and canal walls (Figure 5).

## 4. Discussion

Application of root canal sealers with appropriate properties such as adhesion, adaptation, and tubular penetration is very important for successful RCT. The present* in vitro* SEM study not only comprehensively compared the tubular penetration of four major types of root sealers in the coronal, middle, and apical parts, but also evaluated their adaption capacity. The maximum tubular penetration was exhibited by RealSeal SE, followed by AH-Plus, iRoot SP, and Cortisomol. As regards the adaptation property, AH-Plus has best adaptation capacity followed by iRoot SP, RealSeal SE, and Cortisomol.

For the tubular penetration depth, our results showed that RealSeal SE penetrated deepest in the dentin tubule compared with the other three sealers. RealSeal SE is the fourth-generation methacrylate resin-based sealer and several possible reasons might account for its better performance. First, the addition of dilute into RealSeal SE not only reduces resin viscosity but also increases the flow of the self-adhesive resin monomers. In addition, the incorporation of 4-methacryloyloxyethyl trimellitate anhydride (4-META) into the sealer can make it self-etching and hydrophilic. The self-etching feature enables the sealer to penetrate the smear layer and partially demineralize the normal dentin beneath the hybrid layer, which can clean and enlarge the openings of the dentin tubules. The hydrophilic property of 4-META makes the sealer adapt to the collagen fiber network and penetrate through the hybrid layer as well as the demineralized dentin to enter a deeper depth in the dentin tubules [11–13]. AH-Plus is epoxy-resin-based sealer and its flow might be as good as RealSeal SE. Epiphany SE has the same components as RealSeal SE and Resende et al. reported that AH-Plus and Epiphany SE were similar in terms of flow [12]. However, AH-Plus penetration was much shallow compared with RealSeal SE. Lack of diluted resin, acid monomer, and hydrophilic monomer in AH-Plus might reduce its permeability into the dentinal tubules. iRoot SP is a bioceramic-based sealer composed of biocompatible nanosphere components [14]. Our results revealed that iRoot SP had similar tubule penetration depth in the coronal and middle third of the root canals in comparison with AH-Plus, indicating that this sealer also has relatively good permeability. Nanoparticle based structure can increase the flow property and surface activity of iRoot SP. In addition, the reaction between resin matrix and calcium phosphate forms packing-like substance, which has good flow property [15]. Its hydrophilic components also enable the sealer to enter into the dentin tubule smoothly [16]. The penetration depth of iRoot SP was significantly decreased in the apical third. One possible reason was the complex apical anatomy. In addition, the presence of sclerotic, transparent, and occluded dentin in the apical third of root canals might also remarkably reduce the permeability of iRoot SP as it does not contain acidic monomers. The traditional sealer Cortisomol had the poorest performance. The particles absorb the water and trigger spontaneous aggregation, which will then reduce the surface energy, increase the adhesion capacity, and decrease the flow capability of the sealer. Therefore, the highly flowable, self-etching, and hydrophilic properties of RealSeal SE are the major reasons for its deepest tubular penetration depth among the four sealers. AH-Plus and iRoot SP also have good permeability. However, they penetrated less depth compared with RealSeal SE due to lack of acidic monomers.

The epidemiology has showed that apical microleakage is the major reason for RCT failure and the microgap between the sealer and root canal walls leads to apical microleakage. Consistent with previous study [17], the results showed that AH-Plus had good adaptation property and the gaps between AH-Plus and canal walls were less than 1 *μ*m. The shrinkage of AH-Plus is relatively small because of its good flow property; thereby AH-Plus can form homogenous, regular, and streamline chemical adhesion with root canal walls. The adaptation of RealSeal SE was worse than AH-Plus and the gap between RealSeal SE and canal walls ranged from 1 *μ*m to 10 *μ*m in most cases. Several reasons might be responsible for the moderate performance of RealSeal SE. First, the humidity at the apical portion and the natural residue moisture not only affect the conversion efficiency of RealSeal SE, but also promote the hydrolysis reactions of methyl methacrylate resulting in crack formation. Moreover, the polymerization shrinkage of RealSeal SE also causes gap between sealer and canal walls. iRoot SP has similar adaptation capacity to root canal walls compared with RealSeal SE. The smear layer in the apical area and deficiency of acidic monomers in iRoot SP limit its sealing ability. Large gaps (>10 *μ*m) were observed between Cortisomol and canal walls. The surface of Cortisomol is porous after it is set, which makes it difficult to generate smooth form with intertubular dentin and peritubular dentin. In addition, its bonding with canal walls is nonchemical; thus the sealer is vulnerable to detach from root canal walls [18].

One potential limitation of the current study was that our results were entirely based on the observation under SEM. Micro-computed tomography (micro-CT) was also effective for the evaluation of voids and gaps formation [19, 20]. Thus combination of SEM and micro-CT might provide a more accurate finding for assessing the adaptation capacity of root canal sealers. Recently some novel bioceramic-based sealers have shown great potential due to their physical and biological properties such as alkaline pH, chemical stability within the biological environment, and lack of shrinkage. In addition, they contain calcium phosphate which contributes to the formation of the crystalline structure, which can significantly improve the sealer-to-root dentin bonding [21, 22]. Future studies should evaluate the tubular penetration depth and adaptation of these relatively novel sealers.

In conclusion, the data demonstrated that RealSeal SE has deepest tubular penetration depth and AH-Plus has best adaptation property. The permeability and adaptation ability of iRoot SP is moderate compared with the above two resin-based sealers. We do not recommend Cortisomol for RCT due to its poor performance.

## Figures and Tables

**Table 1:** The penetration depth and gap width of four sealers (*μ*m).

	*n*	Coronal	Middle	Apical
Gutta-percha/Cortisomol	12	33.41 ± 8.55	11.67 ± 4.92	5.13 ± 2.43
Gutta-percha/iRoot SP	12	66.30 ± 24.46	25.68 ± 11.01	6.78 ± 3.00
Gutta-percha/AH-Plus	12	69.82 ± 21.79	26.13 ± 11.81	19.10 ± 7.87
Resilon/RealSeal SE	12	114.10 ± 26.25	42.82 ± 13.14	31.93 ± 10.86

**Table 2:** The width of gaps between the sealers and the apical third of the root canal walls (*μ*m).

Group	Apical
Gutta-percha/Cortisomol	25.62 ± 8.54
Gutta-percha/iRoot SP	6.86 ± 2.87
Gutta-percha/AH-Plus	0.97 ± 0.43
Resilon/RealSeal SE	6.46 ± 1.60

## References

[1] Haapasalo M., Udnaes T., Endal U. (2003). Persistent, recurrent, and acquired infection of the root canal system post-treatment. *Endodontic Topics*.

[2] Love R. M., Jenkinson H. F. (2002). Invasion of dentinal tubules by oral bacteria. *Critical Reviews in Oral Biology and Medicine*.

[3] Schilder H. (2006). Filling root canals in three dimensions. 1967. *Journal of Endodontics*.

[4] Muliyar S., Shameem KA., Thankachan RP., Francis PG., Jayapalan CS., Hafiz KA. (2014). Microleakage in endodontics. *Journal of International Oral Health*.

[5] Marciano M. A., Guimaraes B. M., Ordinola-Zapata R. (2011). Physical properties and interfacial adaptation of three epoxy resin-based sealers. *Journal of Endodontics*.

[6] Kim Y. K., Grandini S., Ames J. M. (2010). Critical review on methacrylate resin–based root canal sealers. *Journal of Endodontics*.

[7] Gesi A., Raffaelli O., Goracci C., Pashley D. H., Tay F. R., Ferrari M. (2005). Interfacial strength of Resilon and gutta-percha to intraradicular dentin. *Journal of Endodontics*.

[8] Wu M. K., De Gee A. J., Wesselink P. R. (1998). Effect of tubule orientation in the cavity wall on the seal of dental filling materials: an in vitro study. *International Endodontic Journal*.

[9] Sjögren U., Figdor D., Persson S., Sundqvist G. (1997). Influence of infection at the time of root filling on the outcome of endodontic treatment of teeth with apical periodontitis. *International Endodontic Journal*.

[10] Heling I., Chandler N. P. (1996). The antimicrobial effect within dentinal tubules of four root canal sealers. *Journal of Endodontics*.

[11] Babb B. R., Loushine R. J., Bryan T. E. (2009). Bonding of Self-adhesive (Self-etching) Root Canal Sealers to Radicular Dentin. *Journal of Endodontics*.

[12] Resende L. M., Rached-Junior F. J. A., Versiani M. A. (2009). A comparative study of physicochemical properties of AH plus, epiphany, and epiphany SE root canal sealers. *International Endodontic Journal*.

[13] Moura-Netto C., Palo R. M., Camargo S. E. A., Jent C., Leonardo R. D. T., Marques M. M. (2012). Influence of prior 810-nm-diode intracanal laser irradiation on hydrophilic resin-based sealer obturation. *Brazilian Oral Research*.

[14] Uzunoglu E., Yilmaz Z., Sungur DD., Altundasar E. (2015). Retreatability of root canals obturated using gutta-percha with bioceramic, MTA and resin-based sealers. *Iranian Endodontic Journal*.

[15] Özcan E., Çapar İ., Çetin A. R., Tunçdemir A. R., Aydinbelge H. A. (2012). The effect of calcium silicate-based sealer on the push-out bond strength of fibre posts. *Australian Dental Journal*.

[16] Sagsen B., Üstün Y., Pala K., Demirbuğa S. (2012). Resistance to fracture of roots filled with different sealers. *Dental Materials*.

[17] Cavenago B. C., Duarte M. A., Ordinola-Zapata R., Marciano M. A., Carpio-Perochena A. E., Bramante C. M. (2012). Interfacial adaptation of an epoxy-resin sealer and a self-etch sealer to root canal dentin using the System B or the single cone technique. *Brazilian Dental Journal*.

[18] Upadhyay V., Upadhyay M., Panday R. K., Chturvedi T. P., Bajpai U. (2011). A SEM evaluation of dentinal adaptation of root canal obturation with GuttaFlow and conventional obturating material. *Indian Journal of Dental Research*.

[19] Hammad M., Qualtrough A., Silikas N. (2009). Evaluation of root canal obturation: a three-dimensional in vitro study. *Journal of Endodontics*.

[20] Wolf M., Küpper K., Reimann S., Bourauel C., Frentzen M. (2014). 3D analyses of interface voids in root canals filled with different sealer materials in combination with warm gutta-percha technique. *Clinical Oral Investigations*.

[21] Poggio C., Dagna A., Ceci M., Meravini M., Colombo M., Pietrocola G. (2017). Solubility and pH of bioceramic root canal sealers: a comparative study. *Journal of Clinical and Experimental Dentistry*.

[22] Polineni S., Bolla N., Mandava P., Vemuri S., Mallela M., Gandham V. M. (2016). Marginal adaptation of newer root canal sealers to dentin: a SEM study. *Journal of Conservative Dentistry*.

